# Case report: Metreleptin rapidly improved anorexia nervosa related and comorbid psychopathology in a patient with high endogenous leptin levels adjusted for body mass index

**DOI:** 10.1007/s00787-025-02809-3

**Published:** 2025-07-05

**Authors:** Jochen Antel, Gertraud Gradl-Dietsch, Triinu Peters, Lutz Pridzun, Franziska Degenhardt, Linda von Piechowski, Anke Hinney, Johannes Hebebrand

**Affiliations:** 1 Department of Child and Adolescent Psychiatry, LVR-University Hospital Essen, University of Duisburg-Essen, Wickenburgstrasse 21, 45147 Essen, Germany; 2 Section for Molecular Genetics of Mental Disorders, University Hospital Essen, Essen, Germany; 3 Center for Translational Neuro- and Behavioural Sciences, University Hospital Essen, Essen, Germany; 4 Institute of Sex- and Gender-Sensitive Medicine, University Hospital Essen, Essen, Germany; 5 Mediagnost, Gesellschaft für Forschung und Herstellung von Diagnostika GmbH, Reutlingen, Germany

**Keywords:** Leptin, Anorexia nervosa, Antidepressant, Starvation, Hyperleptinemia, Hypoleptinemia

## Abstract

Off-label treatment of a 17-year-old female patient with anorexia nervosa (AN), major depressive disorder (MDD), obsessive-compulsive disorder (OCD), posttraumatic stress disorder (PTSD), and non-suicidal self-injury (NSSI) with human recombinant leptin (metreleptin) for two dosing periods of 15 and seven days was associated with self- and clinician-rated improvements of eating disorder related psychopathology, OCD, depression, and NSSI. These intermittent improvements occurred despite the patient having high endogenous serum leptin levels ranging up to the 99th centile adjusted for sex, Tanner stage and body mass index (BMI; kg/m²) upon initiation of dosing. The results further extend the hypothesized effects of metreleptin on AN and comorbid psychopathology to patients with adjusted leptin levels in the normal to high range. We hypothesize that for unknown reasons the clinical symptomatology of a subgroup of patients with AN persists despite attainment of endogenous leptin levels well above those characteristic of the state of starvation, potentially entailing a prolonged entrapment in the eating disorder. An insight into the mechanisms underlying this distinct type of leptin resistance may help to promote recovery of such affected patients. Placebo controlled randomized clinical trials are required to assess if and to what extent short and medium term metreleptin treatment indeed improves recovery.

**Supplementary Information:**

The online version contains supplementary material available at 10.1007/s00787-025-02809-3.

## Introduction

AN is characterized (DSM-5) [2] by underweight, fear of weight gain, body image disturbances and behaviours inconsistent with the attainment/maintenance of a normal body weight. Patients frequently have comorbid MDD, which may be induced by starvation [31, 33]. Other comorbid disorders include anxiety disorders [38], NSSI [4] and OCD [11].

Leptin is an adipokine, whose serum concentration is correlated with adipose tissue mass. Congenital deficiency of leptin in mice (*ob/ob* mouse) and humans results in hyperphagia, extreme obesity and infertility [40]. Acquired hypoleptinemia triggers the neuroendocrine adaptation to starvation [26] via the hypothalamic-pituitary-end organ axes. In addition, leptin receptors located both centrally and peripherally enable tissue specific adaptations to subnormal concentrations of leptin [29]. In rodents, leptin deficiency induced by food restriction triggers increased running wheel activity [13]. Acute AN is characterized by hypoleptinemia [15, 22, 24] as a result of the reduced adipose tissue mass. Transient hyperleptinemia can occur upon treatment induced weight gain [24].

We have treated single patients with AN and hypoleptinemia off-label with metreleptin [3, 18, 39]. Substantial improvements were observed in five of six patients treated for between 6 and 24 days. Similarly, a patient with atypical AN and a leptin level in the lowest normal range also improved [30]. Further, a male patient with AN and probable myalgic encephalomyelitis/chronic fatigue syndrome responded favourably despite a leptin level corresponding to the 99th centile adjusted for sex, Tanner stage and BMI [28].

Here, we focus on the off-label metreleptin treatment of a 17-year-old female patient with AN, who had serum leptin levels of 9.1 ng/ml and 11.5 ng/ml prior to two 14- and seven-day long dosing periods separated by 3.5 months.

## Case report

Patient T and her two sisters had postural orthostatic tachycardia syndrome during adolescence. At age 16.1 (see Suppl. Fig. S1) T developed restrictive eating behavior and a pronounced increment in physical activity. BMI at first inpatient admission at age 16.3 years after loss of nine kg was 16.2 kg/m² (≈ 1st age and sex adjusted centile; 49.7 kg; 175 cm). Liver enzymes were slightly elevated, phosphate and erythrocytes reduced. She suffered from constant preoccupation with food and body weight. Therapeutic progress was slow and characterized by a fragile commitment during her second and third inpatient treatments (see Suppl. Fig. S1). Her condition intermittently deteriorated requiring nasogastric feeding. She was almost mutistic due to social phobia; she developed NSSI. After the second inpatient episode she switched to day care treatment, during which she made little progress over 18 weeks. After brief emergency treatment to control hyponatremia, she was readmitted with a BMI of 18.4 kg/m² due to compulsive water drinking of up to twelve litres daily obsessively fearing that harm would otherwise come upon her dizygotic co-twin. Eating disorder cognitions and inner tension were severe. Somatic findings included acrocyanosis, tachycardia (up to 141 bpm) and slightly elevated liver enzyme values. She developed intermittent vomiting and a disgust of looking at or touching food entailing compulsive handwashing lasting for hours. Comorbid DSM-5 diagnoses were OCD, NSSI, insomnia, and MDD. The patient was treated with an exposition therapy with response prevention and fluvoxamine resulting in less compulsive drinking. Concomitantly, her eating disorder cognitions worsened. Because of her serious condition she and her parents agreed to off-label treatment with metreleptin despite a high leptin level adjusted for BMI, sex and Tanner stage (d-5: 9.1 ng/ml).

Due to the pronounced overall improvement after a 15 day-long dosing period with metreleptin and the nine-month long inpatient/day care treatment the patient requested to be discharged despite medical reservations as to the medium-term prognosis. Indeed, readmission (BMI 15.9 kg/m²; see fourth treatment episode in Suppl. Fig. S1) was required four weeks later due to a renewed weight loss of six kg, daily compulsive water drinking of 7.5 L, increased self-injurious behaviour, depression and hyperactivity (30,000 steps/d according to her step counter). She had again developed extensive hand washing fearing contamination and uptake of food. Physical examination revealed scratching, rubbing, razorblade cutting, and hand dermatitis. Leptin level was 7.1 ng/ml upon readmission. Nasogastric feeding was reinitiated in light of insufficient weight gain. During the seven day long second metreleptin dosing period the patient again improved. She for the first time reported having witnessed a sexual abuse of a friend at age nine. She developed infrequent flashbacks; diagnostic criteria for post-traumatic stress disorder (PTSD) were met. She was able to achieve a weight of 62 kg (19.6 kg/m²; height 178 cm) at discharge.

Two months after discharge she had again lost weight (BMI 15.4 kg/m²), which led her to seek inpatient treatment at a psychosomatic hospital for three months. She regained weight to a BMI of 18.2 kg/m², but suffered from excessive hyperactivity (40,000 steps: seven hours of rapid walking daily including one hour during nighttime), three hours of handwashing, poor concentration precluding school attendance, insomnia and eating disorder specific cognitions. She contacted her health insurance to request reimbursement of a longer-term metreleptin treatment without success (see Suppl. text 2). Nevertheless, her condition improved slowly; she was able to maintain her weight at approximately 55 kg for one year, found a boy-friend and completed school. After the final exams she again lost weight (15.3 kg/m²) and is currently (two years after last dosing episode) being treated as an inpatient in a psychosomatic hospital. She is convinced that despite a slow recovery she is off the danger list. In retrospect, she views metreleptin treatment as providing her with continuous hope that her eating disorder is surmountable and as having cured her social phobia. She has continuously been amenorrheic throughout the duration of her eating disorder.

## Methods

Patient and parents agreed to short-term off-label metreleptin treatment (written informed consents) added to long-term fluvoxamine (50-0-75 mg during both dosing periods). During the second dosing period, sodium glycerophosphate (d-10 to d6) and pantoprazole against heartburn (d-10 to d + 10) represented additional medications. Psychoeducation focused on expected metreleptin-induced clinical changes, endocrine adaptation to starvation, and necessity to use boost in motivation to gain weight to normalize endogenous leptin secretion.

### Dosing periods

Metreleptin was applied subcutaneously at 9:00 am (dosing period 1: 3 mg: d1, d2, d5, d10, d12, d15; 5.8 mg: d3, d4, d6-d9, d11, d13-d14; dosing period 2: 5.8 mg: d1-d6; 3 mg: d7). Emotions and cognitions were tracked with visual analogue scales (VAS; daily means of morning and evening scores) [3, 18, 39]. Clinician and self-ratings were obtained with Children’s Depression Rating Scale-Revised (CDRS-R) [32] and German versions of Beck Depression Inventory-II [21], and Eating Disorder Inventory-2 [46]. Serum leptin levels were measured at 9 am (prior to application of metreleptin). Oral food intake was complemented with nasogastric feeding during the second dosing period (330–660 kcal/d).

### Determination of serum leptin concentrations and sequencing of leptin gene

Two serum samples were measured in parallel with both Mediagnost kit E077 SENSITIVE Leptin for immunoreactive leptin (irLEP) and kit L07 for functional Leptin (bioLEP) two and eight weeks after the second dosing period to evaluate receptor binding. E077 measures immune-reactive leptin using two monoclonal antibodies, both binding to leptin and thereby generating the measured value (sandwich antibody binding). Kit L07 quantifies only functional leptin binding to the soluble leptin receptor detected via an antibody. For non-modified leptin, both test methods give identical results (quotient bioLEP/irLEP = 1).

Genomic sequences of LEP gene (two coding exons, Chromosome 7: 128,241,278 − 128,257,629, GRCh38.p13) were extracted from the Ensembl Database (http://www.ensembl.org/index.html). The cDNA and protein sequence of the transcript variant 1 (LEP-201, ENST00000308868.5) were used. Primer pairs were designed using online software PRIMER3 (https://primer3.ut.ee/). Primers were analyzed using BLAST (https://blast.ncbi.nlm.nih.gov/Blast.cgi) of UCSC Genome Browser to verify the designed primer’s specificity. Polymerase chain reaction (PCR) amplified DNA samples were sequenced by Microsynth Seqlab GmbH (Göttingen, Germany). All sequences were analyzed using the SeqMan Pro software by DNAStar, Inc. (version: 10.1.0) and evaluated by two (AH and coworker) experienced scientists.

## Results

**Fig. 1:**
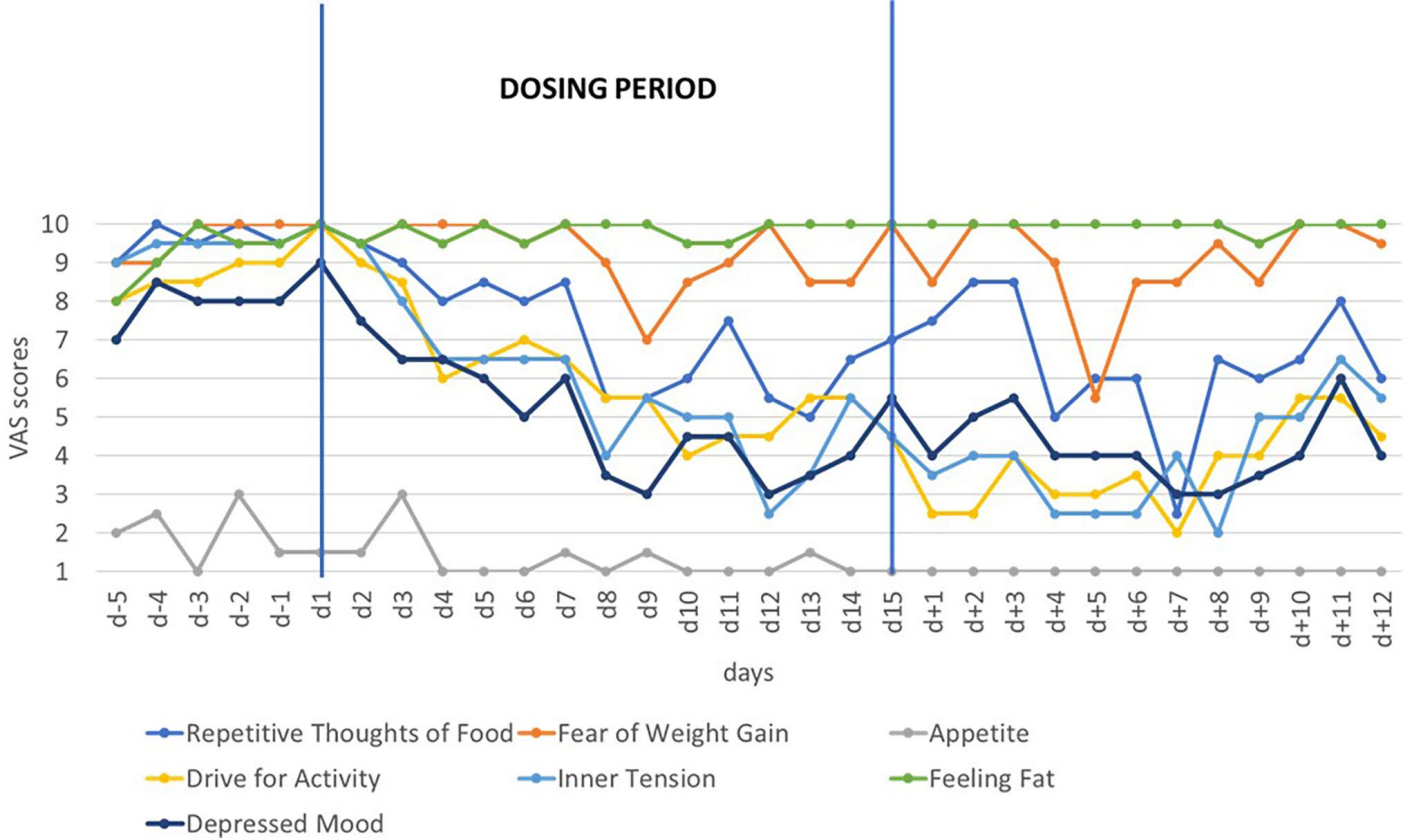
Means of self-rated eating disorder and mood associated cognitions and emotions assessed twice daily with visual analogue scales (range 1–10) prior to, during and after the first 15-day dosing period

During the first dosing period, eating disorder related psychopathology began decreasing within one to two days (Fig. 1). Clinically relevant reductions in CDRS-R and BDI-II [9, 16, 35, 42] were observed. The BDI-II score dropped from 53 (d-5) to 39 at d + 9 (see Supplementary Fig S2). The patient reported improved sleep quality, less tiredness, exhaustion and mood swings. The compulsive symptomatology including urges to harm herself, hand washing (decreased feeling of disgust when seeing and touching food), water drinking, and vomiting decreased. Additionally, preoccupation with the eating disorder was reduced and conversely the motivation for recovery increased. The clinical improvement was noticeable to family members and staff. However, VAS items ‘feeling fat’ and ‘fear of weight gain’ (Fig. 1) and EDI-2 raw scores (Suppl. Table 1) did not change over time. After cessation of dosing, sleep quality declined and heart palpitations increased again (Fig. 3a, b); other improvements persisted for a few weeks. During this time period she viewed herself as for the first time wanting to leave a barren room to which she had withdrawn (see Suppl. Text 1). The second dosing period, too, was associated with a rapid and intermittent improvement of her condition.

**Fig. 2:**
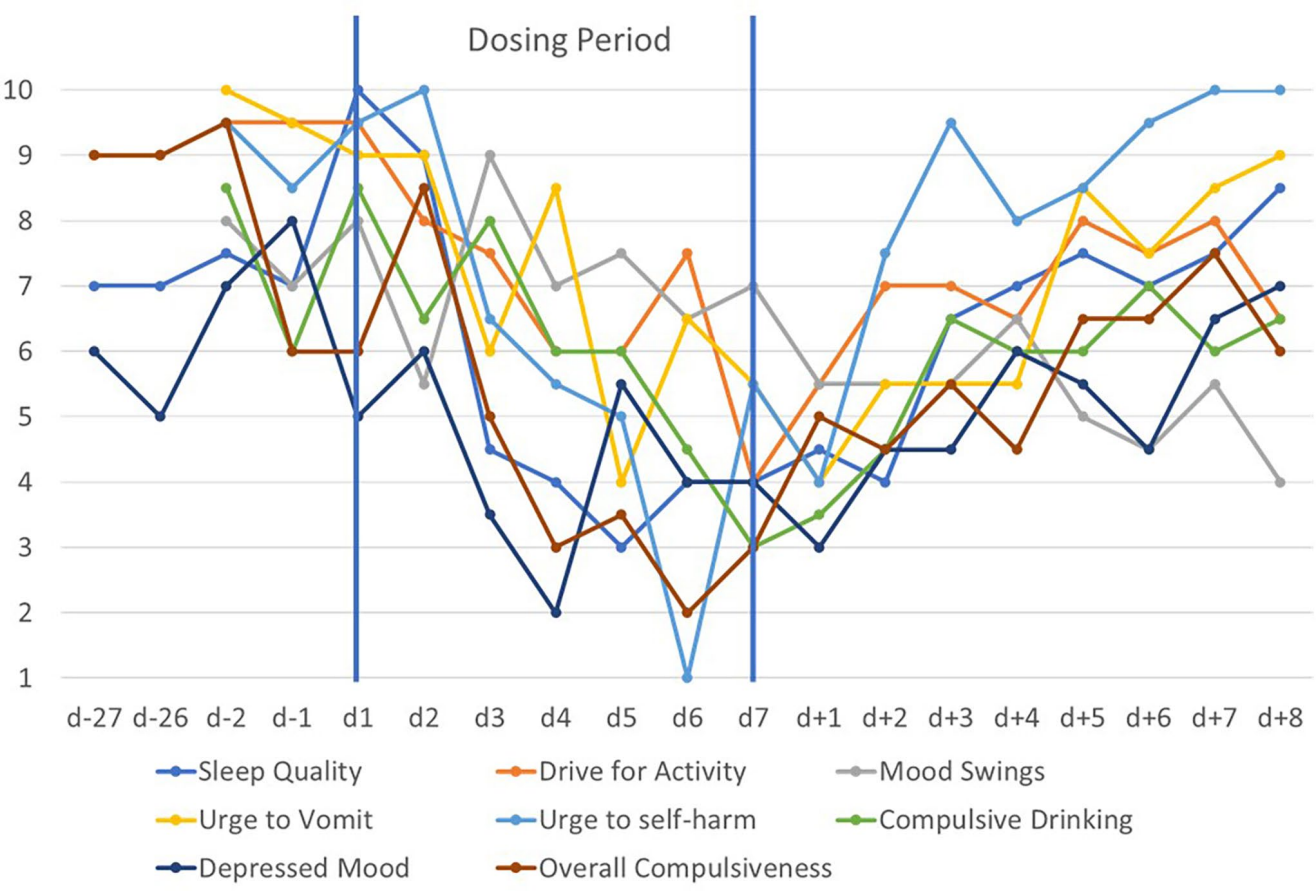
Means of self-rated mood related cognitions, emotions and obsessions/compulsions assessed twice daily with visual analogue scales (range 1–10) prior to, during and after the second 7-day dosing period

**Fig. 3:**
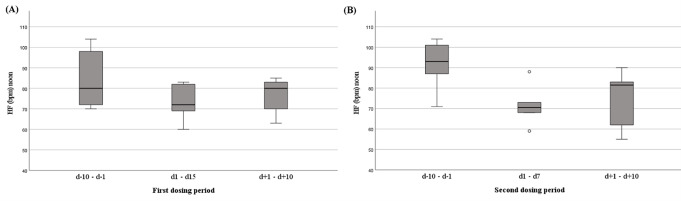
**A and B**: Boxplots showing transient effects on mean pulse rates (beats per minute (bpm)) prior, during and after the two dosing periods

Circulating leptin levels were unexpectedly high (9.1 ng/ml; >99th percentile adjusted for BMI, sex and Tanner stage [6] five days prior to treatment (d-5) and stayed high (9.0 ng/ml at d6) during dosing. Measured levels during dosing comprised both exogenous and endogenous leptin; due to the half-life of 3.8–4.7 h for metreleptin [14], blood samples obtained prior to dosing should mainly contain endogenous leptin.

During the second dosing period, AN related key cognitions again began dropping within 1–2 days (compare Figs. 1 and 2). Rapid improvements for the VAS items sleep quality, mood swings, urge to vomit, urge to self-harm, compulsive drinking, and overall compulsiveness (incl. excessive hand washing) were observed (Fig. 2). The feeling of disgust upon seeing and touching food also decreased again. The CDRS-R score fell from 69 at d-1 to 57 at d6. BDI-II (Fig. S2) scores dropped from 47 (d-6) to 38 at d6 remaining at the same level at d + 3 and rebounding to 46 at d + 9. Raw total score of the EDI-2 (64 items) [46] dropped slightly from 244 at d-5 to 213 at d6 to rebound after end of dosing (Supplementary Table S1). After cessation of dosing and similar to the first post-dosing period, sleep quality deteriorated and heart palpitations increased again (see Fig. 3); other positive changes persisted for a few weeks only. During dosing, the patient reported the “opportunity to briefly distance myself from my problems” (see Suppl. Text 2). Additionally, motivation for recovery increased, noticeable to herself, family members and treatment staff. She was able to gain weight up to 62.2 kg six weeks after the end of dosing period two.

### Heartbeat and blood pressure

In contrast to commonly AN associated hypotension and bradycardia [8, 44] T had high blood pressure and tachycardia prior to both dosing periods (tachycardia predated initiation of fluvoxamine treatment). Surprisingly, pulse rate normalized during dosing (Fig. 3a, b) and rebounded upon cessation.

### Body weight

Medium-term trends in body weight change were not altered by the two metreleptin dosing periods (Suppl. Figure 1). Weight declined minimally during first dosing period and increased during the second.

### Serum leptin

**Fig. 4:**
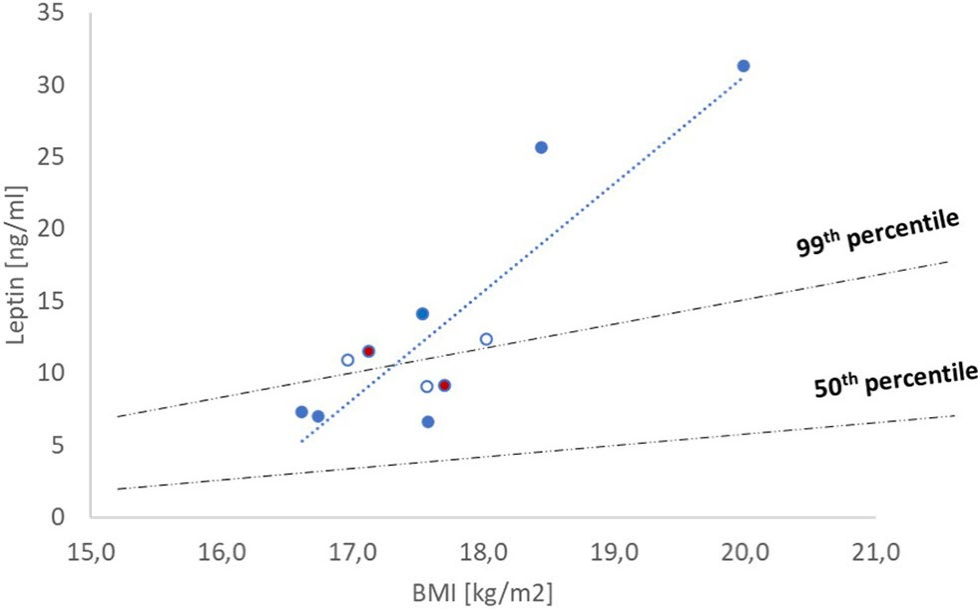
BMI and leptin values recorded over half a year prior to, during and after the two dosing periods. Open circles indicate values measured during the first and second dosing periods. Leptin levels where 9.1 ng/ml (55.1 kg) prior to the first dosing (d-5) and 11.5 ng/ml (53.3 kg) prior to the second dosing period (d-2); respective datapoints are indicated in red. Dotted line = trendline showing the extraordinary steep slope between BMI and leptin levels for patient T. The two dotted-dashed lines show the 50th and 99th percentiles for leptin levels [6] adjusted for BMI, sex, and Tanner stage based on a reference sample of healthy adolescents girls [6]

Prior to both dosing periods, T had normal or high levels of leptin adjusted for BMI, sex and Tanner stage (Fig. 4; reference sample based on healthy female adolescents [6]). BMI and leptin levels of the patient were correlated (Fig. 4), but the slope was steep with levels surpassing the 99th adjusted percentile [6]. In both samples taken prior to dosing, the immune-reactive leptin values were lower than the corresponding values for functional leptin (bioLEP/irLEP quotient > 1.0) [48]. The sequencing of the leptin gene revealed no mutations.

## Discussion

This sixth case report of off-label metreleptin treatment of AN recapitulates previous beneficial mental and behavioural changes [3, 18, 39] and confirms (see [27]) clinical amelioration despite normal (dosing period 1) and exceedingly high (dosing period 2) baseline leptin levels adjusted for BMI, sex and Tanner stage [6]. During dosing, pronounced improvements in comorbid OCD, NSSI, and emotional instability were observed; tachycardia normalized. After cessation of 15- and seven day-long dosing periods, her condition deteriorated, more rapidly after the shorter second dosing period. Similar to the previous case reports we cannot exclude placebo effects. For example, promising initial results for intranasal application of oxytocin were not confirmed in a recent double blind randomized controlled trial [37].

The apparent persistence of eating disorder specific cognitions and emotions and comorbid disorders despite normal or high endogenous leptin levels adjusted for sex, Tanner stage and BMI is reminiscent of the widely used but inadequately defined terms “clinically observed leptin non-responsiveness” [41] or “leptin resistance” [17] in the context of obesity. The assessment of determinants of clinical leptin responsiveness is hampered by a lack of robust, easily quantifiable behavioural or metabolic biomarkers of leptin’s action [41]. In acute AN, hypoleptinemia [23, 24] due to loss of adipose tissue entails the neuroendocrine adaptation to starvation [5, 25, 47]. Patient T had already gained weight prior to the first dosing period, thereby likely accounting for her high normal leptin secretion. Clearly in contrast to classical leptin resistance in obesity [41], the metreleptin induced boost in leptin signalling was seemingly able to induce similar improvements as previously observed in patients with hypoleptinemia. We speculate that (a) the metreleptin boost in leptin signalling overcomes a reduced responsiveness to endogenous leptin, (b) unknown peripheral mechanisms entail a lack of biologically active leptin, and (c) because of the attainment of high leptin levels during metreleptin treatment [3, 39] a bolus effect may be required to induce clinical improvements. The range between serum leptin levels of 2 to 4 ng/ml has been shown to differentiate patients with acute AN from healthy constitutionally underweight females [15, 34].

The amelioration of OCD, NSSI and emotional instability are remarkable and speak against expectation effects, which nevertheless cannot be excluded [39]. Similarly, delusional thoughts related to ingestion of food via touching also rapidly declined during dosing entailing less compulsive hand washing. The rapid reduction of perceived inner tension may speculatively be responsible for reduced urge to self-harm; improved sleep quality is also compatible with a pronounced reduction of inner tension. Likewise, the lowered heartbeat rate may reflect the strong reduction in inner tension, which is known to entail high pulse rates in susceptible individuals [45]. A direct effect of metreleptin also appears possible. Thus, circulating leptin levels may centrally mediate blood pressure and heart frequency [10, 19] or even locally via leptin receptors located in sinus node and/or atrial and ventricular myocytes [36]. Hyperleptinemia is assumed to play a causal role in the development of obesity-associated hypertension [1, 20]. On the other hand, in hypoleptinemic patients with AN, mean systolic blood pressure values are significantly lower in comparison to controls [43].

In contrast to previous case reports [18, 39] the improvements during dosing were more strongly time limited (Fig. 2). Patient T was clearly improved in the initial weeks after the dosing periods but in the medium term became ‘entrapped’ again. Her description of wanting to leave a room into which she had withdrawn (possibly as a result of PTSD) provides a glimpse into her mental condition (Suppl. Text 1). The patient was keen to be again treated with metreleptin hoping that a longer dosing period would allow her to better pursue recovery (see her reimbursement request for a medium-term treatment episode; Suppl. Text 2). After having been continuously ill for two years after the second dosing episode the condition of the patient improved allowing her to start a three-year training program as an occupational therapist. The patient stated that metreleptin cured her social phobia; the positive response enabled her to develop the hope that her eating disorder will be surmountable. For obvious reasons, the investigators are unable to assess if the transient improvements observed during the dosing periods influenced the course of the eating disorder. Similar to the thoughts of the patient, we can only speculate that either longer term treatment or more frequent shorter episodes might have sped up her recovery process.

In this context, it is important to consider side effects of metreleptin. In patients with generalized lipodystrophy, these potentially include the development of antibodies that neutralize endogenous leptin and/or metreleptin, lymphoma, hypoglycaemia, autoimmunity and hypersensitivity [12]. Because generalized lipodystrophy is in itself associated with an elevated risk of lymphoma, it is currently unclear if the occurrence of lymphoma in single metreleptin treated patients is due to metreleptin (one-armed clinical trial for the orphan disease generalized lipodystrophy) [7]; to our knowledge lymphoma has not been observed in small numbers of metreleptin treated patients with other diagnoses (e.g. hypothalamic amenorrhea, congenital leptin deficiency). The very common occurrence of hypoglycemia in patients with generalized lipodystrophy and diabetes warrants close monitoring of patients with AN, particularly upon initiation of dosing. We have only treated inpatients for whom we were able to ensure a sufficient daily caloric intake; care must be taken to assure that food is ingested several times a day orally or via nasogastric feeding and that weight loss does not occur. Weight loss has been observed in patients with lipodystrophy and hypothalamic amenorrhea after medium term treatment for four weeks. Local reactions at the injection site occur in a subgroup of patients, which may necessitate stopping the treatment. Acute pancreatitis has occurred after discontinuing metreleptin treatment of patients with lipodystrophy. Hypersensitivity including anaphylaxis has been reported in patients with lipodystrophy who had been treated for several weeks (for a complete overview of side effects see [12, 14] and [25]. Because metreleptin may reduce the adaptation to starvation, only medically stabilized patients should be considered for this treatment. In conclusion, the occurrence of potentially dangerous side effects requires their assessment in placebo controlled trials for patients with AN [25]. In light of the very limited knowledge, we strongly recommend inpatient treatment and appropriate monitoring for off-label application of metreleptin [12].

Under the given assay conditions and reaction equilibria upon the twofold measurement of leptin levels, a similarly elevated value for bioLEP/irLEP has been observed in patients with leptin mutations [48]. However and as expected, sequencing of the leptin gene of patient T revealed no mutations. Upon further dilutions of the two samples, higher absolute values for leptin were found in both assays, each increasing further with higher dilution. Because of a stronger relative increase of the measured value for irLEP, identical values were observed upon the final dilution (bioLEP/irLEP = 1). This observation may indicate the existence of an interfering factor, which upon dilution no longer influences measurements in both test systems.

In conclusion, our results support and widen the medical hypothesis that a substantial proportion of the symptomatology of AN results from leptin deficiency either by the “usual”– starvation related - depletion of endogenous leptin or an extraordinary type of “leptin resistance”. We again question the primary role of leptin as an anorexigenic hormone [18] and strengthen the hypothesis that both central and peripheral effects of leptin extend well beyond the established neuroendocrine adaptation via the hypothalamus-pituitary-end organ axes [26, 29].

Clinically, severely and desperately ill patients with AN may profit from off-label leptin treatment; unfortunately, the high drug costs represent a major impediment. Finally, clinical trials are warranted to substantiate and potentially expand these initial findings. Further research is required to elucidate the mechanisms underlying high bioLEP/irLEP quotients.

## Electronic supplementary material

Below is the link to the electronic supplementary material.

Supplementary Material 1

## Data Availability

All directly sharable data are supplied with the supplementary material.
