# Supplementary material for: Case report: Metreleptin rapidly improved anorexia nervosa related and comorbid psychopathology in a patient with high endogenous leptin levels adjusted for body mass index
Source: Eur Child Adolesc Psychiatry. 2025 Jul 5;34(10):3307–15. doi: 10.1007/s00787-025-02809-3 (PMC12592268; doi:10.1007/s00787-025-02809-3)
Supplement: Supplementary file 1 — Supplementary Material 1 [file 787_2025_2809_MOESM1_ESM.docx]

## **Case Report:** **Metreleptin rapidly** **improved anorexia nervosa related and comorbid psychopathology in a patient with high endogenous leptin levels adjusted for body mass index**

Jochen Antel^1,3^, Gertaud Gradl-Dietsch^1^, Triinu Peters^2,3,4^, Lutz Pridzun^5^, Franziska Degenhardt^1^, Linda von Piechowski^1^, Anke Hinney ^2,3,4^, Johannes Hebebrand ^1,3^

1. Department of Child and Adolescent Psychiatry, LVR-University Hospital Essen, University of Duisburg-Essen, Essen, Germany
2. Section for Molecular Genetics of Mental Disorders, University Hospital Essen, Essen, Germany.
3. Center for Translational Neuro- and Behavioural Sciences, University Hospital Essen, Essen, Germany.
4. Institute of Sex- and Gender-Sensitive Medicine, University Hospital Essen, Essen, Germany.
5. Mediagnost, Gesellschaft für Forschung und Herstellung von Diagnostika GmbH, Reutlingen, Germany

Supplementary Material

**Supplementary text 1: “The barren room”;** written from patient T in German and translated with www.DeepL.com/Translator (free version)

It is an empty room in a large busy room, although it is in the middle of the house, it is like quite far away. The room has only one big heavy door and one window, but you can hardly see out of it because the shutters are down, you can only see out through some small gaps.

The room is completely empty, no furniture, no electricity, just a concrete floor and white walls, nothing spectacular really.

This room is a retreat, initially. When I first entered the room, I didn't think how much it would shape me and my life one day, it was only for a short time and it gave me peace, I could do my thing without anyone judging me. The room was a kind of release from all the social stress that was outside of it, so I used the room more and more and more, someday I closed the door, all the social pressure, everything has to be perfect, the comparison between people, just too much, I used the space. The closed door gave me the security that no one could also take away my space. The room became my room, not only my room, but my world, my life somehow also. In the beginning I still looked out of the window or the shutter gaps and looked, from time to time I opened the door, but I could never leave the room.

I heard noises from outside for a while, but I could not really perceive them. I got used to this room more and more, I didn't want to leave it anymore. I neither looked out of the window, nor did I open the heavy door, whereby I lacked the strength anyway.

One day people tried to open the door from the outside, but it was stuck, they asked me to help to open the door to the hallway in the big room again, but I didn't want to and couldn't, no one was allowed to take my room away from me, it was all I had by now, I had gotten too used to it, but somehow I lost myself in this room.

Again, and again people tried to open the door to get me back into the house and free me from the room, they needed my help, but I just couldn't do it. It all seemed unsuccessful.

A new method (*reference to off-label metreleptin treatment*) came to get me through the window. For the first time after such a long time, I looked out between the gaps of the shutters. I saw life, friends and future, but somehow, I didn't care. I saw people trying to help me, but something stopped me. At some point I managed to open the shutters completely, I was overwhelmed and shocked at the same time. I saw what was outside the room again after such, such a long time. I saw life as I knew it from before, but I also lived in my room, I was torn. Which of these is the real life?

More and more I discovered and observed life outside, I began to like it, I wanted this life too.

Getting out of this room was my greatest wish, but the door and the window were both closed.

The life outside encouraged me, I managed to get up from the concrete floor, I went to the window, the window openers were locked. It took me some time to find an idea I could try to open the window. I took a piece of broken concrete, no matter how heavy it is, I carry it on with the motivation that I turn into strength, until I can finally break the windowpane with it, but it is not so easy, the concrete is heavy, the doubts are great, and the room is my actual life that I am supposed to leave behind. But is it worth it? Why can't I just walk out the door and call it a day? Why does it come to so many difficulties?

**Supplementary text** **2: Excerpt of the patient’s written request for reimbursement of metreleptin treatment;** written from patient T in German and translated with www.DeepL.com/Translator (free version)

I received my first leptin dose on December 16, 2020, at which time I was already in the LVR Klinikum in Essen in the Department of Child and Adolescent Psychiatry, Psychosomatics and Psychotherapy for my anorexia nervosa for the second time after almost a year of inpatient and partial inpatient therapy.

First of all, I would like to mention that I have been carrying symptoms of anorexia nervosa for about three years. Initially, this manifested itself in excessive sports behavior and a diet, which I strictly followed. But as time went by, more and more problems developed. My head circled all day long only around the topic of food and weight loss. I planned every meal several times and always checked everything very carefully before I ate anything. In parallel, the number of calories I ate went down in 200kcal steps until I only ate one apple a day. As if I wasn't already busy enough with weighing and calculating meals and their calories and thinking about different foods, I developed a strong feeling of disgust towards any thought of eating. So, if I even saw something edible, a very strong feeling of disgust spread through me, but also a great fear that I could gain weight by looking at or smelling the food. Touching food became especially bad for me. It triggered such a strong feeling of disgust in me that I had to wash my hands and arms for hours afterwards. The fear of having absorbed calories by touching the food spread through my whole body, so that I could not do anything else but wash my hands for hours until they were red and sore. But even that was often not enough. The tension caused by the feeling of disgust and the fear of having taken in calories triggered great dissatisfaction and self-loathing in me, which I could not regulate down. It is a terrible feeling when you can't stand yourself in your own skin and hate yourself so much that it led to self-injurious behavior. In states of tension, I often scratch all over my arms without really noticing it. I was in such a state of tension that I neither realized what I was doing there, nor did I feel any pain. Since this also hardly reduced my tension, I began to scratch and cut myself with razor blades, with each time it became deeper and deeper, so that I had to be treated by trauma surgery several times. Even then, I felt no pain in the high tension.

As if all this wasn't enough and took up enough time in my life that I would have liked to use differently, my anorexia told me to move a lot and do a lot of sports. In my worst phase I walked 30,000 steps a day and in parallel did fitness exercises several times a day to lose as much weight as possible in a short time. Thus, the night was also filled with exercise. So, I didn't sleep properly, but spent the nights walking in circles around my room for up to four hours, collecting my steps.

So weeks passed without sleep, so that during the day I could barely keep my eyes open from fatigue, but the inner turmoil was so great that resting or taking a break was not an option at all. During the day I spent time at school, it was terrible. Sitting in a chair for hours and listening to one person and not losing focus. It was impossible for me. The school hours felt like school weeks. With tears in my eyes, I sat down on the chair in the morning at school, immediately my legs started bouncing. My head told me I couldn't just sit and do nothing. Thoughts shot through my head, "Get up, you can't sit. You'll get fat, really fat.' I couldn't stand it. I had to leave class several times. There, the tension often reached its peak. I had to walk in circles, count my steps. But even that was no longer enough at some point. I was overcome by the thought of vomiting for the first time, even though I didn't even have any food in my stomach, since I only ate something small in the evening. Nevertheless, I saw no way out and began to vomit several times a day (up to 9 times) both at school and at home.

I noticed how I was getting worse from day to day, both physically and mentally. This quickly became apparent in my social life as well. I no longer managed to see my friends or family because I was simply too preoccupied with the disease. Getting up every day became a torture, everything hurt, I was constantly dizzy, and I was cold all day, no matter how thickly I dressed. With the last of my strength, I went to school every day. But even that got worse and worse, so that I felt so bad that I fell asleep at school from fatigue.

My friends and family noticed that I was not well and increasingly spoke to me about my condition, but I refused any help. Even though I was miserable, seeing the weight go down was such a great feeling. It felt like a huge success that made me feel like I was doing something very well. But that feeling only ever lasted a short time, followed very quickly by thoughts of not being good enough, which again reinforced the self-hatred, which again led to a lot of self-harming behavior.

All this robbed me of any possible positive feelings and thoughts. I despaired more and more and felt alienated from myself, as if I could no longer keep myself under control.

The negative thoughts eventually turned into thoughts about death. I imagined how I could kill myself. I saw no other way out, I was getting worse and no matter how much I did for the anorexia, it was never good enough, which put me permanently under high tension.

The high tension also affected me physically. My blood pressure and pulse rates went through the roof, permanently I felt my heart racing. No matter how hard I tried to regulate myself down and relax, my tension kept rising and rising.

I came acutely the first time in the LVR clinic in Essen with judicial order, because the anorexia had me at that time so strongly in the grip, so that I believed her and did not want to let her go. At the clinic I was force-fed, told about the risks of anorexia, and shown how harmful my situation was. But I couldn't understand all that, I wanted to keep losing weight because I was good at it. The weight gain that came from the probing didn't totally destroy me mentally. The self-loathing increased with every gram more and I hurt myself more and more and deeper. After the court order expired, I let myself be discharged with the idea that if I lost weight again, that I would get better. I spent four months in a day clinic lying to everyone and telling them that I was trying to get better. In the background there was always the thought that as soon as I have the chance to lose as much weight as possible again. During the time I was in the day clinic, I gained about 3kg. This made me very mentally exhausted, so that I continued to self-harm and wash my hands for up to 2-3 hours after every meal that I was forced to eat. When in the course of the day clinic treatment my mother got breast cancer, I lost all motivation to get better. The thought ', it must not go badly for others, it must go badly for me' bored itself firmly into my head. The fear that others could feel bad rose very high in a short time, especially with my family. I then developed a water-drinking compulsion, which was supposed to weaken obsessive thoughts such as "your sister will feel bad, she will get sick". In a short time, this compulsion became so strong that I drank up to 12L of water a day to reduce the obsessive thoughts. The day hospital could no longer handle the responsibility and thus I was discharged. One week later I was again acutely admitted to the LVR clinic in Essen, there I was deprived of any possibility even to get water. Not to have the possibility to follow his compulsion, released to all my already high tension and inner restlessness only more of it. I was given medication to ease my compulsions and reduce the tension, but it just didn't help.

One day I got the offer that I could try the drug Leptin, after much deliberation I decided to do so and I am still grateful to have had this opportunity.

I received my first dose of Leptin in December 2020, when I had already spent a month in inpatient treatment for obsessive-compulsive disorder and anorexia, but there had been no great improvement in my symptoms up to that point.

From about the third day of the leptin dose, I noticed the first effect. I noticed that my motor skills became calmer, and my inner restlessness decreased, and I felt a pleasant tiredness for the first time in a long time. Over the next few days my sleep improved so that I could sleep up to 8 hours a night, mostly without interruption, which meant that I finally felt refreshed after all this time. I got the feeling that I was able to concentrate better and that my attention was also increased again. Every day I decided to read something and day by day I read more and more pages in one go. I developed interest in old positive activities again, such as my horse. I hadn't taken care of it for months because I had no interest or energy to clean or feed it. So, I was able to enjoy the time with my horse very much. I also noticed my family more and started spending time with them again. Instead of being in my room and running in circles, I baked with my family for Christmas and cuddled with my cat, which I could perceive completely differently than before. It felt like everything that used to be normal was special and I was happy about little things that I hadn't even noticed before. So, the time I spent doing nice activities, I saved on steps. My step count was reduced by almost half, from 30,000 steps a day to 15,000-18,000 steps a day. I also consciously sat down several times a day for half an hour instead of standing around or exercising. I started writing to my friends over and over again during this time and spending time with them, which I hadn't done for months. It made me so incredibly happy to have my family and friends around that I cried a lot with joy. It's hard to describe how good I was feeling at that point, because it was really, really good. Still, there were many more beautiful moments. Since my anorexia illness, I never sat down at the piano again, which was actually my old hobby. But one day I sat down in front of my parents at the piano in the clinic and played them a Christmas song. It was an incredibly beautiful and liberating moment. I can no longer imagine my life without actively playing the piano. I went through so many beautiful moments in such a short time, which I still carry in my heart today and which also encouraged me a lot to fight the anorexia. It is as if I had been replaced, my motivation was at a maximum at that time, and I gave all my best to defeat the disease. Getting healthy again was my greatest wish at that time, taking my life was not an option for me anymore, because I really enjoyed that time. I wanted to defeat this stupid disease with the greatest will.

I also felt increasingly better physically. The sleep made me feel more refreshed the next day and fit for the day, but also my pulse and blood pressure, which were otherwise elevated, normalized and the constant heart palpitations also disappeared, and I felt relaxed.

Because the motivation for recovery was very high, I was able to gain weight quickly during the leptin treatment, so that I was able to gain 12kg during the leptin and post-treatment phases, thus normalizing my weight. The feeling of disgust when seeing and touching food also decreased. I managed not to wash my hands after eating but could use this time to relax. There was also a feeling of strength and "I can do this". When the leptin treatment ended after 15 days, I noticed a decline in the positive effects, sleep worsened and the heart palpitations also increased again, but the other effects continued for a few more weeks, so that I felt fitter and happier and continued to participate in the therapy with motivation until the symptoms worsened again.

Since the first leptin treatment was so successful, we started a second leptin trial in March 2021, which went for seven days. This one also quickly improved my general condition, making me feel fitter for recovery and very motivated to continue working on my problems. The motivation that I developed through the leptin-trial was stronger than ever before, because experiencing a better situation gave me more and more the will to finally get well again. I was given the opportunity to briefly distance myself from my problems and thus regain time and interest in enjoyable activities. Even in retrospect, I like to think back to this beautiful time, in which I was really doing very well.

Today, unfortunately, I no longer have any effect of the medication, the thoughts of food and the associated feeling of disgust circle in my head all day long. The urge to move also deteriorated greatly, so that I now have to walk about 30,000 steps a day again and sitting down is also a great torture for me. The other positive effects of the leptin have also disappeared, so I feel similar to how I did before the leptin.

Also, my weight, which I was able to keep in a healthy weight range during the leptin devotion, is currently back to underweight and despite efforts, unfortunately, no matter how hard I try, it is not possible for me to gain my own weight into a healthy weight range in this condition.

However, thanks to the great experience with Leptin, I already have many fond memories of the time when I was better. I would love to get back to this point and be completely healthy again, but despite any attempts, this is simply not possible for me without the drug Leptin.

**Supplementary Figure S1:**  Body weight of patient T over a period of 114 weeks beginning with the first inpatient treatment period. Orange boxes indicate in-patient treatment episodes; measured body weights outside of these boxes were derived from outpatient visits. The two dosing periods are indicated. Recalled pre-morbid weight eight weeks prior to the initial presentation was 58kg.

_
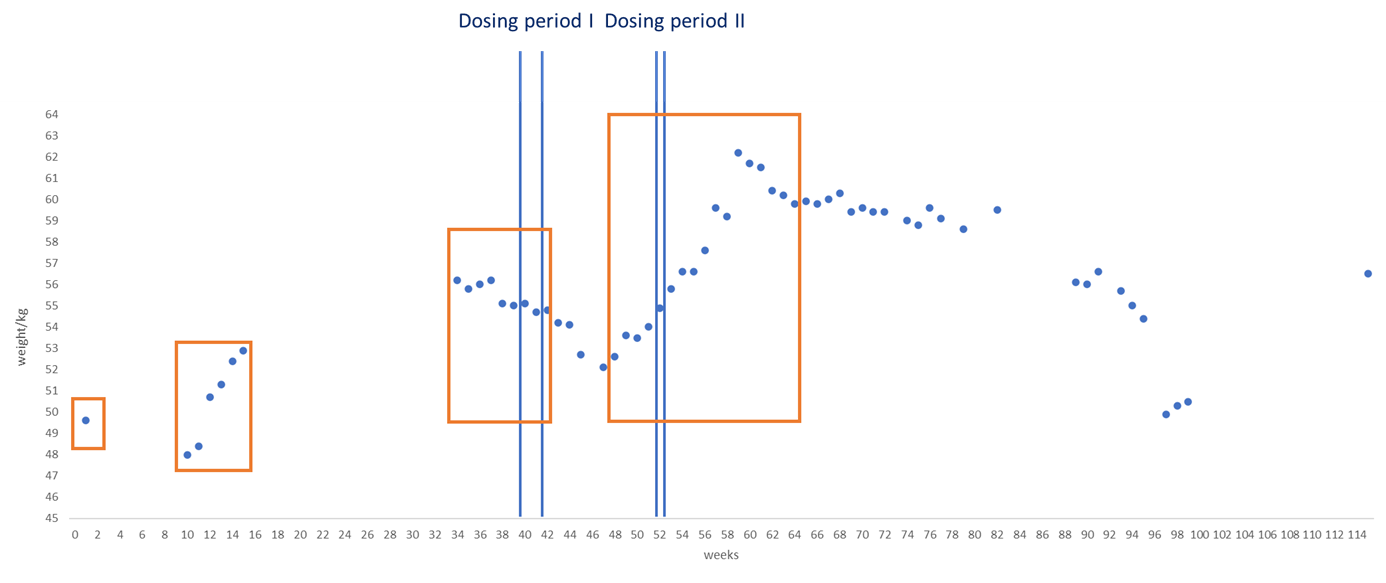
_


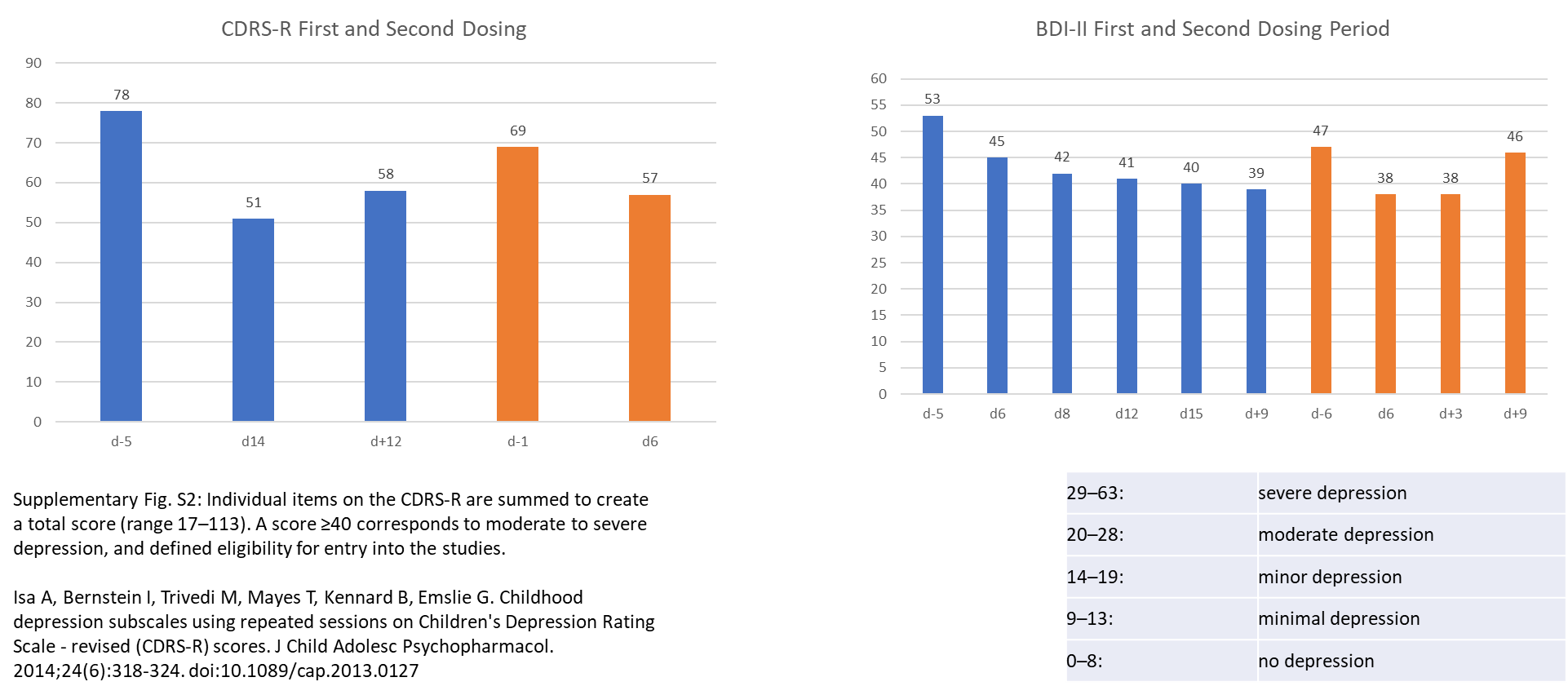


Supplementary Table 1
